# Novel *KIAA0825* Variants Underlie Nonsyndromic Postaxial Polydactyly

**DOI:** 10.3390/genes16091118

**Published:** 2025-09-21

**Authors:** Abdullah, Thashi Bharadwaj, Saffia Javed, Hammal Khan, Anushree Acharya, Weizhen Ji, Umm-e-Kalsoom, Hamid Ali, Isabelle Schrauwen, Wasim Ahmad, Saquib A. Lakhani, Suzanne M. Leal

**Affiliations:** 1 Department of Biochemistry, Faculty of Biological Sciences, Quaid-i-Azam University, Islamabad 45320, Pakistanwahmad@qau.edu.pk (W.A.); 2 Center for Statistical Genetics, Gertrude H. Sergievsky Center, Department of Neurology, Columbia University Medical Centre, New York, NY 10032, USA; 3 Department of Biochemistry, Hazara University Mansehra, Mansehra 21120, Pakistan; saf.javed11@gmail.com (S.J.); kalsoom.u@hu.edu.pk (U.-e.-K.); 4 Department of Biosciences, COMSATS University, Islamabad 45550, Pakistan; hkhan@bs.qau.edu.pk (H.K.);; 5 Department of Translational Neurosciences, University of Arizona College of Medicine-Phoenix, Phoenix, AZ 85004, USA; 6 Pediatric Genomics Discovery Program, Department of Pediatrics, Yale University School of Medicine, New Haven, CT 06510, USA; 7 Department of Pediatrics, Cedar-Sinai Medical Center, Los Angeles, CA 90048, USA; 8 Taub Institute for Alzheimer’s Disease and the Aging Brain, Columbia University Medical Center, New York, NY 10032, USA

**Keywords:** exome and Sanger sequencing, *KIAA0825*, nonsyndromic autosomal recessive postaxial polydactyly (PAP), PAP type A and type B

## Abstract

Background: Extra digits on the hands and/or feet are a frequent condition known as polydactyly. Twelve nonsyndromic polydactyly genes have been identified, including *KIAA0825*. Methods: Four consanguineous Pakistani families that segregate nonsyndromic postaxial polydactyly (PAP) with an autosomal recessive mode of inheritance were clinically and genetically evaluated. Exome sequencing or genotyping of polymorphic microsatellite markers followed by Sanger sequencing were used to identify the variants underlying the PAP etiology. Results: Three novel *KIAA0825* variants were identified that segregate with PAP: a nonsense variant c.2319G>A; p.(Trp773*) in two families; a missense variant c.970G>T; p.(Val324Phe) in one family; and a four amino acids in-frame deletion c.2743_2754del; p.(Gln915_Val918del) in one family. The nonsense variant segregated in families with PAP type B (PAPB), while the missense and the in-frame deletion variants segregated with PAP type A and B. Conclusions: The findings of this study expanded the clinical and genetic spectrum of PAP due to *KIAA0825* variants including the first *KIAA0825* variant specific to PAPB.

## 1. Introduction

Polydactyly is characterized by the presence of extra fingers or toes and is the most common congenital disorder of the hands and feet. Polydactyly occurs more often in upper than the lower limbs and on the right side than the left [1,2]. Polydactyly can occur either due to defects in programmed cell death or in the signaling pathways involved in the anterior–posterior patterning of developing fetal limbs. There are both nonsyndromic and syndromic forms [3,4]. Although over 200 genes have been identified for syndromic polydactyly, only 12 genes have been reported for nonsyndromic polydactyly: *DACH1* (MIM:603803); *EFCAB7* (MIM:617632); *FAM92A/CIBAR1* (MIM:617273); *GLI1* (MIM:165220)*; GLI3* (MIM:165240); *IQCE* (MIM:617631); *KIAA0825* (MIM:617266); *MIPOL1* (MIM:606850); *PITX1* (MIM:602149); SHH (MIM:600725); *STKLD1* (MIM:618530); and *ZNF141* (MIM:194648).

There are several forms of polydactyly, including postaxial, preaxial, central, and very rare forms, e.g., mirror image, Haas-type polysyndactyly. Postaxial polydactyly (ulnar/fibular), the most common form, is an extra fifth digit [5,6,7,8]. Subclassifications of postaxial polydactyly include type A (PAPA) and the less severe type B (PAPB). In PAPA, articulation of a fully developed supernumerary digit occurs either with fifth metacarpal or metatarsal or with a duplicated metatarsal or metacarpal, while for PAPB the extra digit is rudimentary [9,10].

Preaxial (radial/tibial) polydactyly, the second most common form, is a duplication of the first digit (thumb/toe). Central polydactyly (mesoaxial) is a duplication of the second, third, or fourth digits. Approximately twice as many males as females have polydactyly [11].

The intricate process of human limb development is controlled both spatially and temporally by a complex network of molecular pathways. The limb bud grows along a three-dimensional axis that runs from anterior to posterior, dorsal to ventral, and proximal to distal [1,12]. PAP, which can occur as an individual limb malformation or as a component of a pleiotropic syndrome, can originate from dysregulation in the anterior–posterior patterning of the developing limb. The genetic foundation of PAP is significantly correlated with genes that are more specifically associated with the development of the anterior–posterior axis [12,13]. Several intricate cellular pathways, including hedgehog, WNT, and bone morphogenetic proteins (BMPs), control how human limbs develop [14]. A complete understanding of the genetic machinery underlying limb development and anterior–posterior patterning is lacking and needs to be investigated.

In the present study, we investigated four consanguineous Pakistani families segregating nonsyndromic PAP and identified *KIAA0825* variants which have not previously been reported to be involved in polydactyly: nonsense variant p.(Trp773*) in two families, missense variant p.(Val324Phe) in one family, and a four amino acids deletion p.(Gln915-Val918del) in an additional family.

## 2. Materials and Methods

### 2.1. Recruitment of Families

This study was performed according to the Declaration of Helsinki protocols and approved by the Institutional Review Board (IRB) of Quaid-i-Azam University (IRB-QA-176), Islamabad, Hazara University, Khyber Pakhtunkhwa, Pakistan (IRB-No.73/HU/ORIC/2021/808), and Columbia University Medical Center (IRB-AAAS3421), New York, NY, USA. Written informed consent was obtained from all participating members over the age of 18. Parents provided written informed consent for their children who were less than 18 years of age at the time of study. For children over the age of eight years, assent was obtained. Written consent was also obtained to include photographs of the hands and feet of affected individuals.

A field survey for families affected with polydactyly/skeletal phenotypes was conducted throughout Pakistan. After obtaining signed consent, medical and family history was obtained from the adult study participants. Pedigree drawings were constructed based upon information provided by adult family members. Family members with polydactyly underwent medical examinations to rule out syndromic forms of disease. Radiography of the affected family members was performed at a local government hospital. Photographs of their hands and feet were also obtained.

### 2.2. Blood Collection and DNA Extraction

**Figure 1:**
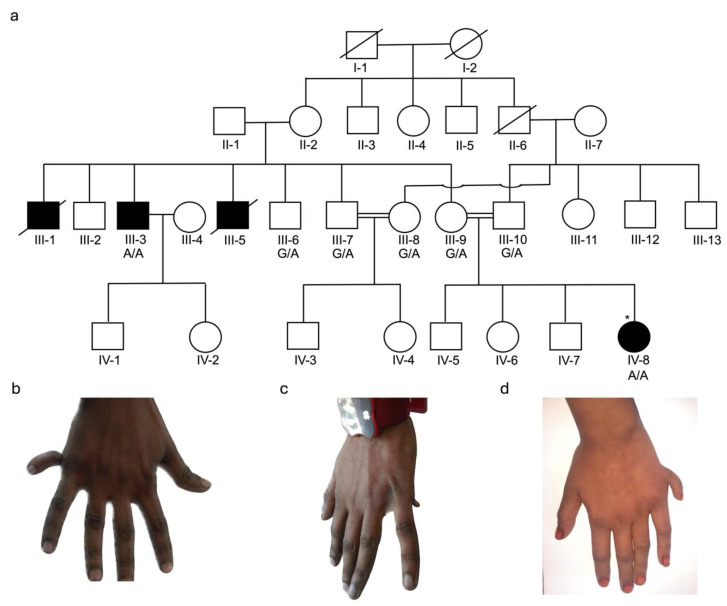
Pedigree BD375 and clinical features for affected family members. (**a**) Pedigree drawing for family BD375 segregating PAPB. Squares represent males and circles females. Double lines indicate known consanguineous relationships and slash lines indicate deceased individuals. Filled symbols represent family members with PAPB and clear symbols unaffected family members. A star (*) indicates the family member whose DNA sample underwent exome sequencing. The genotype for the segregating stop gain variant c.2319G>A; p.(Trp773*) in *KIAA0825* for each family member with an available DNA sample is shown below their identification number. (**b**) Right hand of III-3 displaying PAPB with a superfluous hanging sixth finger. (**c**) Left hand of III-3 with PAPB with a minute outgrowth of supernumerary sixth digit, digit clinodactyly, hand symmetry and digits identity (third, fourth, and fifth) are inarticulate. (**d**) Left hand of IV-8 displaying PAPB, which was the only limb displaying PAP.

**Figure 2:**
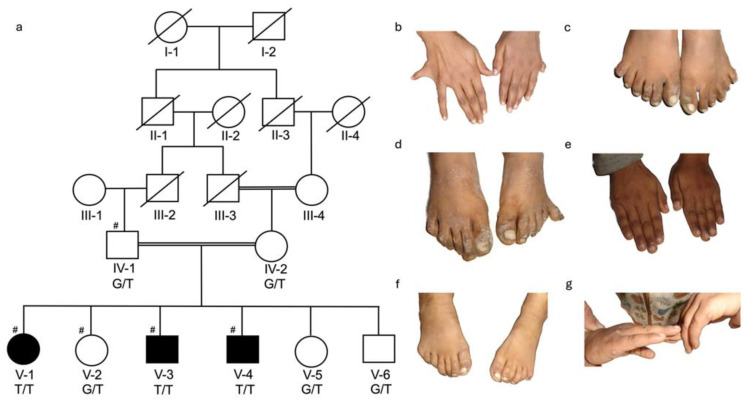
Pedigree BD551 and clinical features for affected family members. (**a**) Pedigree drawing for family BD551 segregating PAPA and PAPB. Squares represent males and circles females. Double lines indicate known consanguineous relationships and slash lines indicate deceased individuals. Filled symbols represent family members with PAP and clear symbols unaffected family members. A number sign (#) indicates the family members whose DNA samples were genotyped. The genotype for the segregating missense variant [c.970G>T; p.(Val324Phe)] in KIAA0825 for each family member with an available DNA sample is shown below their identification number. (**b**) V-1 displays PAPA in her right hand, PAPB in left hand; and (**c**) PAPA in her right and left feet. (**d**) V-3 had PAPB in his right foot which was surgically removed and PAPA in the left foot. (**e**) V-3 has unaffected hands. (**f**) V-4 has feet displaying bilateral PAPA with dysplastic nails of the sixth toes. (**g**) V-4 also had bilateral PAPB of the hands, which were surgically corrected.

**Figure 3:**
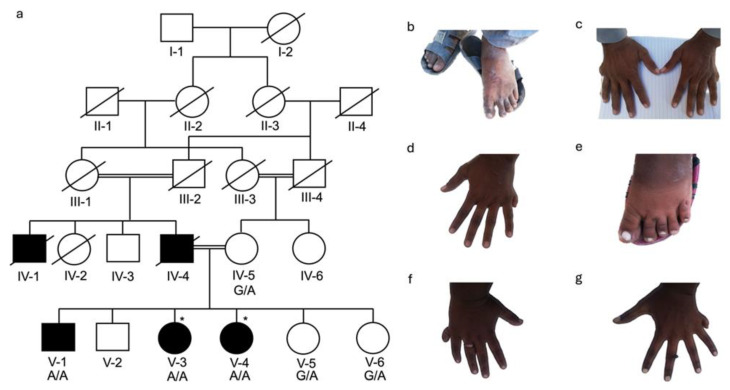
Pedigree BD650 and clinical features for affected family members. (**a**) Pedigree drawing for family BD650 which segregates PAPB. Squares represent males and circles females. Double lines indicate known consanguineous relationships and slash lines indicate deceased individuals. Filled symbols represent family members with PAPB and clear symbols unaffected family members. A star (*) indicates family members whose DNA samples underwent exome sequencing. The genotype for the segregating stop gain variant c.2319G>A; p.(Trp773*) in KIAA0825 for each family member with an available DNA sample is shown below their identification number. (**b**) V-1 has normal feet with whitish toenails. (**c**) Hands of V-1 with bilateral PAPB and whitish fingernails. (**d**) V-3 has PAPB restricted to the left hand with whitish nails. (**e**) Left foot of V-4 with brachydactyly and whitish nails, she had PAPB which was surgically corrected, she also has brachydactyly and whitish nails of the right foot and PAPB which was surgically corrected. (**f**) Left hand of V-4 with PAPB and whitish nails (**g**) Right hand of V-4 with PAPB and whitish nails.

**Figure 4:**
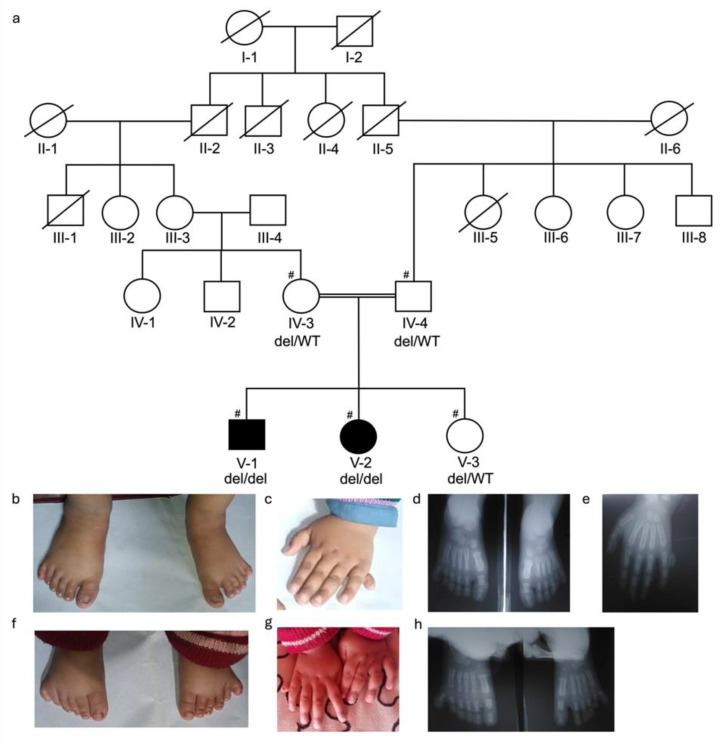
Pedigree KA21 and clinical features for affected family members. (**a**) Pedigree drawing for family KA21 that segregates PAPA and PAPB. Squares represent males and circles females. Double lines indicate known consanguineous relationships and slash lines indicate deceased individuals. Filled symbols represent family members with PAP and clear symbols unaffected family members. A number sign (#) indicates the family members whose DNA samples were genotyped. The genotype for the segregating four amino acid deletion [c.2743_2754del; p.(Gln915-Val918del)] in KIAA0825 for each family member with an available DNA sample is shown below their identification number. (**b**) Feet of V-1 displaying bilateral PAPA originating from fifth metatarsals; (**c**) left hand of V-1 has unilateral PAPB with a rudimentary skin tag; (**d**) X-ray of the feet of V-1 displaying a fully formed extra digit with bony structure that articulates from the fifth metatarsal bone; (**e**) X-ray of the left hand of V-1 which does not display any additional bony structures. (**f**) V-2 with bilateral PAPA in both feet; (**g**) hands of V-2 with unilateral PAPB of the right hand; and (**h**) X-ray of the feet of V-2 displaying a fully formed extra digit with bony structures articulating from the fifth metatarsal.

For DNA isolation, venous blood samples (3–5 mL) were collected in EDTA vacutainers (BD; 10 mL vacutainer, K3-EDTA, Franklin Lakes, NJ, USA) from available affected and unaffected family members. Four families were ascertained, with a total of 26 DNA samples available for study, BD375 (Figure 1: III-3 and IV-8 affected and III-6, III-7, III-8, III-9, and III-10 unaffected members); BD551 (Figure 2: V-1, V-3, and V-4 affected and IV-1, IV-2, V-2, V-5, and V-6 unaffected members); BD650 (Figure 3: V-1, V-3, and V-4 affected and IV-5, V-5, &V-6 unaffected members); and KA21 (Figure 4: V-1 and V-2 affected and IV-3, IV-4, and V-3 unaffected members). Genomic DNA was isolated from whole blood using either standard phenol-chloroform method or commercially available kits (Thermo Fisher, Waltham, MA, USA) and quantified using nanodrop spectrophotometer.

### 2.3. Whole Exome Sequencing and Genotyping

Exome sequencing was performed using DNA samples obtained from an affected member of family BD375 (IV-8) and two affected members (V-3 and V-4) of family BD650 (Figure 1 and Figure 3). Sequencing libraries were prepared using the IDT-xGen capture-kit (IDT, Coralville, IA, USA) or Agilent SureSelect All exon V6 kit (Agilent, Santa Clara, CA, USA). Exome sequencing was performed for BD375 (Figure 1a) and BD650 (Figure 3a) using the Illumina HiSeq4000 and NovaSeq6000 platforms (Illumina Inc, San Diego, CA, USA), respectively. The average exome read depth for BD375 was 100× and for BD650 was 75×. Paired-end sequence reads with low quality were removed and the remaining reads were aligned to the human reference genome (Hg19 for family BD375 and Hg38 for family BD650) using Burrows-Wheeler Aligner-MEM (BWA-MEMV0.7.17) [15]. Duplicate reads were marked using Picard tools(V3.0). Variant calling and base quality score recalibration were performed using the Genome Analysis Toolkit (GATKV4.6.1.0) [16] for single nucleotide variants and insertions/deletions. Variant annotation was performed using ANNOVAR [17], dbscSNV1.1, and dbnsfp35a. In-house custom scripts were used to filter to identify candidate variants. Based on the pedigree structures (Figure 1), since the mode of inheritance was consistent with autosomal recessive for both families, homozygous, or potentially compound heterozygous variants that were exonic, splice region (+/− 12bp), predicted to have an effect on pre-mRNA splicing or protein function (nonsense, missense, start-loss, frameshift, splice region, etc.) with a population specific minor allele frequency (MAF) of <0.005 in the Genome Aggregation Database (gnomAD) [18], were retained.

For families BD551 (Figure 2a) and KA21 (Figure 4a) polymorphic microsatellite markers flanking known nonsyndromic polydactyly genes were genotyped on a subset of available affected and unaffected members [BD551 (IV-1, V-1, V-2, V-3, and V-4) and KA21 (IV-3, IV-4, V-1, V-2, and V-3)] (Supplementary Table S1). Homozygosity mapping was performed using the Homozygosity Mapper2012 [19] to determine which PAP gene was likely to contain the causal variant.

Primers were designed using primer3PlusV3.3.0 and Primer-BLAST. Sanger Sequencing, was performed using the ABI-3730xl analyzer (Thermo Fisher, Waltham, MA, USA), to evaluate all *KIAA0825* (ENST00000682413.1) regions. Using DNA samples from BD551 (V-1) and KA21 (V-2) to test the coding regions of *KIAA0825* (Figure 2) and also using all DNA samples from the four families to test likely causal *KIAA0825* variants for segregation (Supplementary Table S2).

The pathogenicity of the three identified *KIAA0825* variants was classified using the American College of Medical Genetic (ACMG) guidelines.

## 3. Results

### 3.1. Clinical Description of Families

The four consanguineous PAP families came from four regions of Pakistan [Khyber Pakhtunkhwa (BD375), Sindh (BD551), Balochistan (BD650), and Azad Jammu and Kashmir (KA21)]. The clinical manifestations of PAP were variable among and within the four families. Each of the four families is consanguineous with an autosomal recessive mode of inheritance with BD650 displaying pseudodominance (Figure 1, Figure 2, Figure 3 and Figure 4).

Family BD375: A four-generation consanguineous pedigree with seven individuals with available DNA samples including five unaffected (III-6, III-7, III-8, III-9, and III-10) and two affected members [IV-8 (twenty-one years of age) and III-3 (twenty-seven years of age)] that were ascertained and clinically examined (Figure 1a). Two additional affected pedigree members (III-1 and III-3) were deceased at the time of the study and no phenotype data is available. Both IV-8 and III-3 had nonsyndromic PAP, restricted to their upper limbs. The affected male member (III-3) had bilateral upper limbs PAPB (Figure 1b,c). His right hand had PAPB, with a superfluous hanging digit with a mature nail (Figure 1b). He also had a supernumerary digit (PAPB) on his left hand (Figure 1c). Left hand symmetry and digits identity (third, fourth, and fifth) were inarticulate. On the left hand, clinodactyly of the fifth finger was also prominent (Figure 1c). The female affected member (IV-8) only displayed PAPB in her right hand (Figure 1d). Her left hand and lower limbs were unaffected (no photographs available).

Family BD551: A five-generation consanguineous pedigree with three affected [V-1 (15 years of age), V-3 (13 years of age), and V-4 (9 years of age)] and five unaffected (IV-1, IV-2, V-2, V-5, and V-6) members were ascertained for the study (Figure 2a). Affected individual (V-1) displayed bilateral PAP in both of her hands and feet. PAPA was observed in her right hand and both of her feet, while PAPB was observed in her left hand (Figure 2b,c). Affected individual (V-3) had PAPA in his left foot and PAPB in the right foot (Figure 2d). The sixth digit was surgically removed from his right foot. Both hands were unaffected (Figure 2e). Affected family member (V-4) also displayed bilateral PAPA in his feet and bilateral PAPB of the hands (Figure 2f,g). He exhibited dysplastic nails on the extra digits of his feet (Figure 2f). He underwent surgery on his hands to remove the extra digits (Figure 2g).

Family BD650: A five-generation consanguineous pedigree with three affected [V-1 (25 years of age), V-3 (21 years of age) and V-4 (19 years of age)] and three unaffected (IV-5, V-5, and V-6) family members who participated in the study (Figure 3a). Two additionally affected family members were deceased at the time of recruitment: IV-1 died at ten years of age and was the brother of IV-4 and IV-4 the father of affected family members V-1, V-3, and V-4. Affected male family member V-1 presented with bilateral PAPB in both hands. No polydactyly was observed in his feet. Additionally, whitish nails were observed on his fingers and toes. (Figure 3b,c). Affected individual V-3 manifested PAPB, restricted to her left hand (Figure 3d). Affected individual V-4 displayed PAPB in both of her hands and feet and bilateral brachydactyly in her feet (Figure 3e–g). Her extra toes were surgically removed. All affected individuals had whitish nails. Affected individual V-4 was obese but it is unknown if this is related to PAP.

Family KA21: A five-generation consanguineous family with three unaffected (V-3, IV-3, and IV-4) and two affected individuals [V-1 (3 years of age) and V-2 (1.5 years of age)] were ascertained for the study (Figure 4a). Affected member V-1 presented with PAPA in both feet, with a well-formed duplication of the fifth digits (Figure 4b) and unilateral PAPB in his left hand with a rudimentary skin tag (Figure 4c). Radiographic analysis of his feet showed a fully formed extra digit with bony structure that articulated with the fifth metatarsal bone (Figure 4d); no bony structures were visible in the hand radiograph (Figure 4e). Family member V-2 had bilateral PAPA of both of her feet with fully formed duplication of the fifth digit (Figure 4f) and unilateral PAPB with a rudimentary skin tag observed on her right hand (Figure 4g). Radiographic analysis of her feet showed a fully formed extra digit with bony structures articulated with the fifth metatarsal (Figure 4h). Hand radiographs were unavailable.

Teeth, nails, sweating, and hearing were normal in all affected family members. Neurological problems and facial dysmorphism were not observed. All family members who were heterozygous carriers of a pathogenic or likely pathogenic *KIAA0825* variant did not present with PAP.

### 3.2. Identification of KIAA0825 Variants

A homozygous stop gain variant [c.2319G>A; p.(Trp773*)] in *KIAA0825* was observed in both exomes and segregated with PAP in families BD375 and BD650. The variant has a CADD c-score of 40 and is present in South Asians in gnomAD v4 (MAF = 3.75 × 10^−4^), and MAF = 2.03 × 10^−5^ for all populations. In the All of Us research program [20] database the variant is present with a MAF = 1.0 × 10^−6^. This variant is absent from the Trans-Omics for Precision Medicine (TOPMed) Bravo [21] database.

For families BD551 and KA21, homozygosity in affected family members was found in the genotype data of affected members surrounding the *KIAA0825* gene region. Sanger sequencing revealed a missense variant [c.970G>T; p.(Val324Phe)] for family BD551 and for family KA21, a four amino acid deletion [c.2743_2754del; p.(Gln915-Val918del)]. Sanger sequencing confirmed segregation of the identified variants in both families. The variant [c.970G>T; p.(Val324Phe)] is present in gnomAD v4 in South Asians (MAF = 1.17 × 10^−5^), Europeans (non-Finnish) (MAF = 5.99 × 10^−6^), and with a MAF = 5.05 × 10^−6^ for all populations. The deletion [c.2743_2754del; p.(Gln915-Val918del)] is absent from gnomAD v4. Both variants are absent from the TOPMed Bravo and All of Us.

According to ACMG guidelines, the following classification were obtained: nonsense variant c.2319G>A; p.(Trp773*) is pathogenic (PVS1, PP1, PM2); missense variant c.970G>T; p.(Val324Phe) is likely pathogenic (PP1, PM2, PP3); and the in-frame deletion variant c.2743_2754del; p.(Gln915_Val918del) is likely pathogenic (PP1, PM2, PM4) (Table 1).

## 4. Discussion

**Figure 5:**
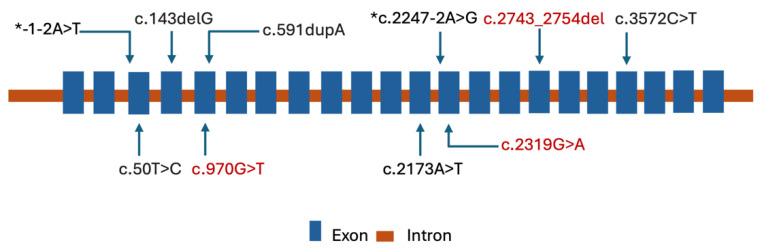
All variants reported in *KIAA0825* in association with PAP visualized using transcript NM_001145678.3. All variants were homozygous except the two variants with a *, which were detected as a compound heterozygous. The seven variants shown in black were previously reported. The three variants in red are the ones presented in the current study.

Previously, five homozygous variants in the *KIAA0825* causing nonsyndromic PAP in the Pakistani population were reported [22,23,24,25]. Additionally, two *KIAA0825* splice site variants in a compound heterozygous state were reported to cause PAPA in a single Chinese patient [26] (Table 2, Figure 5). In this study, we identified three new *KIAA0825* variants underlying nonsyndromic PAP. These were p.(Trp773*), p.(Gln915_Val918del) and p.(Val324Phe). One of the variants, c.2319G>A; p.(Trp773*), which was observed in two families (BD375 and BD650) and caused PAPB and not PAPA. In previously reported families with *KIAA0825* variants, PAPA was always present. It is not clear whether the identification of additional families with c.2319G>A; p.(Trp773*) will confirm that this nonsense variant only underlies PAPB and not PAPA. The *KIAA0825* variants and the resulting phenotypes observed in this, and previous studies are summarized in Table 2 and Figure 5.

Additionally, for the families studied, features in addition to PAP were observed that have not previously been reported to be associated with KIAA0825 variants: brachydactyly (BD650); dysplastic nails (BD551); and whitish nails (BD650).

*KIAA0825*, located on 5q15, has two isoforms: a long isoform (ENST00000682413.1) and a short isoform (ENST00000329378.7), which encode 1275 and 324 amino acids, respectively. The molecular mechanism of *KIAA0825* has not yet been characterized; however, the mouse orthologue (2210408I21Rik) displayed expression in its developing limb buds at E11.5-15.5 and homozygous knockout mice (2210408I21Rik^tm1 (EUCOMM)Wtsi^) had a drastic reduction in bone mineral density [22,26]. The bulk tissue gene expression profile for KIAA0825 shows low-to-moderate expression across most tissues, with the highest median levels observed in whole blood, vagina, small intestine (terminal ileum), and within the brain.

Genetic regulation of molecular pathways in limb development is a complex process that is not fully understood. The marked difference in the number of genes identified for syndromic versus nonsyndromic polydactyly suggests that additional genes and variants are involved in nonsyndromic polydactyly. In this study, we identified three novel variants in *KIAA0825* in unrelated families, strengthening the connection between this gene and polydactyly. However, as the protein remains uncharacterized, its precise role in limb development is difficult to determine. These findings highlight the need for further studies to elucidate the genetic and molecular mechanisms underlying limb formation.

In conclusion, we report three new variants in *KIAA0825* segregating with nonsyndromic PAP. This study expands the clinical and genetic spectrum of the PAPA10 caused by variants in *KIAA0825* and demonstrates that this gene can be responsible for PAPB without PAPA.

## Figures and Tables

**Table 1:** Details on the three novel variants in *KIAA0825* segregating in four families with polydactyly.

Family	Type	Reference Genome	Transcript	Genomic Position (hg38)	cDNA Change	Amino Acid Change	gnomAD All	gnomAD South Asian	Classification ^1^
BD375	Nonsense	Hg19	NM_001145678.3	5:94452997	c.2319G>A	p.(Trp773*)	2.03 × 10^−5^	3.75 × 10^−4^	Pathogenic (PVS1, PP1, PM2)
BD650	Nonsense	Hg38	NM_001145678.3	5:94452997	c.2319G>A	p.(Trp773*)	2.03 × 10^−5^	3.75 × 10^−4^	Pathogenic (PVS1, PP1, PM2)
BD551	Missense	Hg38	NM_001145678.3	5:94520248	c.970G>T	p.(Val324Phe)	5.05 × 10^−6^	1.17 × 10^−5^	Likely pathogenic (PP1, PM2, PP3)
BDKA21	In-frame deletion	Hg38	NM_001145678.3	5:94403701	c.2743_2754del	p.(Gln915-Val918del)	Absent	Absent	Likely pathogenic (PP1, PM2, PM4)

^1^ Classification based on American College of Medical Genetics and Genomics guidelines and the criteria for the classification.

**Table 2:** Description of the variants in *KIAA0825* and the associated PAP phenotypes.

Study	Phenotype	Variant Type	Zygosity	cDNA	Amino Acid Change	Country of Origin
Ullah et al., 2019 [22]	PAPA, PAPB, and clinodactyly	Frameshift	Homozygous	c.591dupA	p.(Gln198Thrfs*21)	Pakistani
Ullah et al., 2019 [22]	PAPA, PAPB and mild camptodactyly	Nonsense	Homozygous	c.2173A>T	p.(Lys725*)	Pakistani
Hayat el at., 2020 [23]	PAPA and camptodactyly	Missense	Homozygous	c.50T>C	p.(Leu17Ser)	Pakistani
Bilal and Ahmad, 2021 [24]	PAPA and syndactyly	Frameshift deletion	Homozygous	c.143delG	p.(Cys48Serfs*28)	Pakistani
Yao et al., 2022 [26]	PAPA	Splice site	Compound heterozygous	c.-1-2A>T	−	Chinese
Yao et al., 2022 [26]	PAPA	Splice site	Compound heterozygous	c.2247-2A>G	−	Chinese
Ahmad et al., 2023 [25]	PAPA, syndactyly, and clinodactyly	Missense	Homozygous	c.3572C>T	p.(Pro1191Leu)	Pakistani
Present Study	PAPB, clinodactyly, brachydactyly, and whitish nails	Nonsense	Homozygous	c.2319G>A	p.(Trp773*)	Pakistani
Present Study	PAPA, PAPB, and dysplastic nails	Missense	Homozygous	c.970G>T	p.(Val324Phe)	Pakistani
Present Study	PAPA and PAPB	In-frame deletion	Homozygous	c.2743_2754del	p.(Gln915-Val918del)	Pakistani

PAP: Postaxial polydactyly; PAPA: PAP type A; PAPB: PAP type B.

## Data Availability

The variants reported in this study were submitted to ClinVar (accession numbers: SCV006084780, SCV006084781, and SCV006084782).

## Supplementary Materials

The following supporting information can be downloaded at: https://www.mdpi.com/article/10.3390/genes16091118/s1, Table S1: List of markers for families BD551 and KA21; Table S2: Primers used to test the segregation of the KIAA0825 variants.
